# Communication is key: combinatorial phosphorylation and functional cross-talk in the RNA polymerase II CTD code

**DOI:** 10.1042/BST20250032

**Published:** 2026-08-07

**Authors:** Y. Jessie Zhang, Erin Ramsey

**Affiliations:** Department of Molecular Biosciences, University of Texas, Austin

**Keywords:** Combinatorial PTM Code, Epitope Masking, RNA Polymerase II CTD, Transcription Elongation

## Abstract

The C-terminal domain (CTD) of the largest subunit of RNA polymerase II serves as a dynamic platform coordinating transcription and RNA processing. Recent advances in mass spectrometry, structural biology, and sequencing approaches have revealed a far more complex, combinatorial CTD code than previously appreciated. The present review examines the CTD as an integrative signaling hub that translates diverse environmental signals into genomic outputs. We explore the hierarchical transitions between phosphorylation states, highlighting how combinatorial marks rather than isolated modifications shape transcriptional progression and prevent cryptic initiation through cross-talk with the histone code. Special focus is given to the roles of ‘enigmatic’ marks such as pTyr1 and pThr4, whose functional significance has been complicated by biochemical masking effects and species-specific regulatory nodes. We also discuss emerging evidence that CTD remodeling contributes to stress-responsive transcription, including oxidative stress, heat shock, and DNA damage. Together, these observations support a dynamic model of the CTD in which combinatorial phosphorylation states coordinate transcriptional progression, genome stability, and stress-responsive gene regulation.

RNA polymerase II (Pol II) serves as the central engine of eukaryotic gene expression, integrating regulatory inputs that coordinate transcription with RNA processing and chromatin dynamics [1,2]. Central to this regulation is the C-terminal domain (CTD) of its largest subunit, RPB1, which consists of tandem repeats of a conserved heptad motif (Y_1_S_2_P_3_T_4_S_5_P_6_S_7_). While repeated 52 times in *Homo sapiens* and 26 times in *Saccharomyces cerevisiae*, its sophistication extends far beyond simple redundancy. Within the heptad, five residues (Y1, S2, T4, S5, and S7) can be phosphorylated, while the remaining residues, P3 and P6, can be isomerized. The repeated heptad accommodates diverse phosphorylation and proline isomerization patterns, creating an unusually versatile regulatory platform [3].

Ser5 and Ser2 phosphorylation have been extensively characterized [3,4], while the regulatory functions of the enigmatic residues Tyr1, Thr4, and Ser7 have only recently come into focus. Although mutational analyses have demonstrated that the phenotypic consequences of altering individual CTD residues vary substantially across species, repeat positions, and the extent of residue replacement, large-scale substitutions and deletions consistently reveal that the overall chemical composition of the CTD is under strong evolutionary constraint, as extensive perturbations frequently compromise transcriptional fitness or viability [5–7].

While early models viewed CTD phosphorylation as a series of discrete, sequentially deposited marks, the turn of the century brought accumulating biochemical and structural evidence supporting a combinatorial CTD code, in which multiple neighboring phosphorylation events collectively determine effector recognition [8]. Our understanding of the physiological role of combinatorial CTD patterns has been hindered by a fundamental technical limitation: antibody-based approaches incompletely detect combinatorial phosphorylation, potentially due to neighboring modifications ‘masking’ antibody epitopes [9]. As a result, the true distribution and function of individual CTD marks *in vivo* may have been substantially underestimated.

CTD functions as a dynamic scaffold that recruits regulatory factors throughout the transcription cycle, with its phosphorylation pattern dictating the timing and specificity of these interactions [1–3]. Beyond serving as a recruitment platform, recent studies suggest that CTD phosphorylation also contributes to the physical organization of the transcription machinery [10–12]. Hypophosphorylated CTD preferentially interacts with the Mediator complex and other components of the preinitiation machinery, whereas progressive CTD phosphorylation weakens these interactions during the transition to productive transcription [10,12,13]. Consistent with this model, the intrinsically disordered CTD undergoes liquid-liquid phase separation *in vitro*, and CTD hyperphosphorylation promotes condensate remodeling during the transition from initiation to productive elongation [10–13].

In the present review, we discuss recent evidence supporting the view that CTD phosphorylation functions through combinatorial states rather than isolated modifications. We first examine how cross-talk among phosphorylation marks shapes transcriptional progression and how technical limitations, particularly epitope masking, have obscured important aspects of the CTD landscape. We then explore how CTD phosphorylation communicates with chromatin through coordinated histone modifications and conclude by discussing emerging evidence that CTD remodeling contributes to transcriptional responses to DNA damage, oxidative stress, and heat shock. Together, these observations support a model in which the CTD functions as a dynamic regulatory grammar integrating transcription, chromatin state, and cellular signaling.

## CTD phosphorylations and the cross-talk among them

### The Ser5-to-Ser2 transition as a combinatorial CTD Code

At the onset of transcription, the CTD must first remain hypophosphorylated to permit assembly of the pre-initiation complex (PIC) [14,15]. In support of this hypothesis, recent cryo-EM structures of yeast and human PICs reveal that CTD interactions with Mediator are mediated predominantly through hydrophobic contacts, suggesting that widespread CTD phosphorylation would be incompatible with stable PIC assembly [16,17]. Shortly after initiation, Ser5 phosphorylation becomes the earliest prominent CTD modification [18].

The initiation of transcription across organisms consistently enters through a highly conserved Ser5 phosphorylation state [3]. A likely explanation arises from the intrinsic substrate preference of CDK7, the kinase subunit of TFIIH that initiates CTD phosphorylation during early transcription on an unmodified CTD substrate [19]. UVPD mass spectrometry analyses of CTD kinase specificity *in vitro* indicate that several transcription-associated kinases, including CDK7, CDK9, CDK12, and CDK13, preferentially target Ser5 [19,20]. This shared initial preference reflects a common substrate-recognition mechanism rather than redundant function: CDK7 exhibits particularly strong specificity toward Ser5 [21], whereas CDK9, CDK12, and CDK13 increasingly phosphorylate Ser2 after prior accumulation of Ser5 phosphorylation [4], suggesting that these kinases' substrate preference shifts depending on the prior phosphorylation state of the CTD. Additionally, recent studies suggest that pSer5 promotes recruitment of Spt6 (Table 1), which in turn facilitates association of PAF1C with elongating Pol II, thereby contributing to the establishment of productive elongation complexes and accumulation of Ser2 phosphorylation across gene bodies [22]. These observations are consistent with a hierarchical CTD phosphorylation model in which early Ser5 phosphorylation primes the CTD for subsequent modification and remodeling during elongation [23].

**Table 1:** Selected examples of CTD-binding proteins evaluated by *in vitro* binding assays with short phosphopeptides

Protein–organism	Preferential binding	Enhances interaction	Weakens interaction	Quantification method	Citations
Spt6–Yeast	pY1	pS2, pS5	n.d.	*In vitro*–FA	[24]
Wdr82–Human	pS5	n.d.	pS2 (weakly attenuates)	*In vitro*–pull down	[25]
Nrd1–Yeast	pS5	pS2	pY1	*In vitro*–FA	[26]
Pin1–Human	pS5	pS2	n.d	*In vitro*–FA	[27]
Set2–Yeast	pS2	pS5	n.d.	*In vitro*–SPR	[28]
Pcf11–Yeast	pS2	n.d.	pY1, pS5	*In vitro*–NMR & FA	[29]
Rtt103–Yeast	pS2, pT4	n.d.	pY1, pS5	*In vitro*–NMR & FA	[29]
ESS1–Yeast	pS5	pS2	n.d.	*In vitro*–NMR, *In vitro*–ITC	[30,31]
SCAF4–Human	pS2	pS5	pS7 (attenuation)	*In vitro*–ITC	[32]
SCAF8–Human	pS2	pS5	pS7 (attenuation)	*In vitro*–FA	[33]
RPRD1A/B–Human	pS2	pS7, K7ac	pS5	*In vitro*–ITC	[34]

** ‘Enhances interaction’ and ‘Weakens interaction’ indicate the effect of an additional neighboring CTD modification relative to the preferred phosphorylation state listed in the preceding column.

In contrast with the early appearance of Ser5 phosphorylation, Ser2 phosphorylation progressively accumulates during productive elongation and becomes enriched across downstream gene bodies [18,35]. Although Ser2 phosphorylation has traditionally been viewed as a late-stage CTD mark, recent studies suggest that the initiation-to-elongation transition involves overlapping pSer5/pSer2 states rather than a simple replacement of Ser5 phosphorylation by Ser2 phosphorylation [36,37]. Early elongation is characterized by coexistence of both modifications, reflecting the activities of CDK9 and CDK12/13 to generate hybrid pSer2/pSer5 CTD states immediately downstream of promoter-proximal pausing [38]. These combinatorial CTD species are actively established during CDK9-mediated pause release and represent distinct intermediates in the transition from promoter-associated to elongation-associated CTD states [20].

It is important to note that CTD-state transitions are not governed by a simple sequential kinase cascade. In addition to its prominent role in Ser5 phosphorylation, CDK7 has been implicated in Ser7 phosphorylation and functions as the CDK-activating kinase for multiple transcriptional CDKs [39–41]. Furthermore, CDK9 and CDK12/13 can generate mixed pSer2/pSer5 CTD states during early elongation [20], and recent studies suggest that activation of CDK12/13 by PAF1C contributes to the establishment of these combinatorial phosphorylation patterns [38]. Although CTD kinases are often discussed in terms of preferred phosphorylation targets, CTD-state transitions are not dictated solely by kinase activity. Multiple phosphatases, including Ssu72, Fcp1/CTDP1, and PP1/PNUTS complexes, contribute to selective removal of CTD phosphorylation marks [42–46]. While important to understanding the dynamics of the CTD, their functions are less well understood due to the extensive compensatory effects upon knockout. In addition, Ess1/Pin1-family prolyl isomerases remodel the *cis*/trans configuration of CTD prolines, influencing both kinase and phosphatase recognition [47]. Consequently, the emergence of Ser2-dominant CTD states likely reflects the combined action of kinase, phosphatase, and isomerase activities rather than a simple sequential phosphorylation pathway.

Importantly, different CTD-associated factors exhibit distinct preferences toward these evolving phosphorylation configurations. Chromatin-associated regulators such as Set2 and the RNA-processing factors SCAF4 and SCAF8 preferentially recognize CTD repeats containing both pSer2 and pSer5 (Table 1), consistent with recruitment during early productive elongation when both marks coexist [48]. In contrast, factors including RPRD1A/B, RPRD2, and Pcf11 preferentially bind Ser2-phosphorylated CTD repeats lacking adjacent Ser5 phosphorylation (Table 1), with pSer5 strongly impairing their association [29,34,49]. Because Ser5 phosphorylation is progressively removed during elongation, these differential binding preferences naturally establish a temporal hierarchy of factor recruitment across the transcription cycle.

Within this framework, hybrid pSer2/pSer5 CTD states may function as transitional signaling platforms that recruit factors required during early elongation, whereas subsequent Ser5 dephosphorylation converts the CTD into a more pSer2-dominant state competent for recruitment of late elongation and termination machinery. Thus, the CTD phosphorylation cycle may encode timing information not simply through the appearance of individual marks, but through the hierarchical succession of combinatorial phosphorylation states. These findings highlight the complexity of CTD-state transitions and suggest that combinatorial phosphorylation patterns contribute to factor recruitment.

### pTyr1 and its cross-talk with Ser2

In contrast with Ser5 and Ser2 phosphorylations, whose roles were appreciated early on [50,51], whether Tyr1 undergoes functionally significant phosphorylation during transcription remained controversial for many years [52]. This is somewhat surprising, given that Tyr1 is one of the most highly conserved residues within the RNA polymerase II CTD heptad repeat throughout eukaryotes, suggesting that it fulfills an evolutionarily important function. However, the abundance and apparent functions of this modification differ markedly across species. These observations raise an important question: whether Tyr1 phosphorylation itself varies considerably among organisms, why has the Tyr1 residue remained so highly conserved?

**Figure 1:**
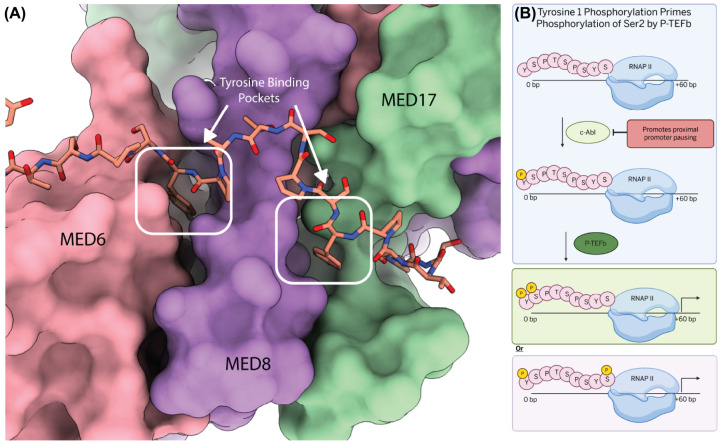
Structural and regulatory roles of Tyr1 in Pol II CTD (**A**) Structural view of the RNA polymerase II CTD bound to Mediator, showing Tyr1 residues inserted into hydrophobic pockets formed by MED6, MED8, and MED17, illustrating the structural basis for Tyr1 conservation. (**B**) Model illustrating Tyr1 phosphorylation as a regulatory node. Tyr1 phosphorylation alters P-TEFb substrate specificity to promote Ser2 phosphorylation of the CTD *in vitro*, potentially to facilitate promoter-proximal pause release and productive transcription elongation.

Structural studies of transcription complexes provide one potential explanation for why Tyr1 itself is essential [16]. In the preinitiation complex, the CTD wraps around the ‘neck’ region formed by the Head and Middle modules of Mediator, where Tyr1 residues insert into hydrophobic pockets that stabilize the CTD–Mediator interface [16]. Mutation of Tyr1 to phenylalanine across more than half of the heptad repeats is lethal [53]. While this phenotype was initially interpreted as evidence that Tyr1 phosphorylation is essential for transcription through its effect on termination [53], structural analyses indicate that the hydroxyl group of Tyr1 participates in hydrogen bonding within the Mediator binding pockets (Figure 1A). Removal of this group weakens the CTD–Mediator interaction and likely destabilizes preinitiation complex formation. These observations suggest that an important component of Tyr1 preservation may derive from its structural role in anchoring the CTD to the transcription machinery.

Importantly, the structural explanation for Tyr1 does not fully account for the diverse phenotypes observed in mammalian CTD mutants. Substitution of Tyr1 can also result in global termination defects [53], altered promoter-proximal polymerase occupancy, and potentially perturbation of Mediator- and Integrator-associated processes [54]. The results indicate that Tyr1 contributes to multiple aspects of transcriptional regulation beyond its role in preinitiation complex architecture. The development of phospho-specific antibodies together with genome-wide analyses established that Tyr1 is phosphorylated *in vivo* and revealed that pTyr1 is enriched near promoters, where it frequently co-occurs with Ser5 phosphorylation [54]. Throughout eukaryotes, Tyr1 phosphorylation has been linked to the regulation of transcription termination. Mechanistically, pTyr1 can impair recruitment of termination factors including Nrd1, Pcf11, and Rtt103 (Table 1) [55], whereas dephosphorylation of Tyr1 near gene ends facilitates engagement of the termination machinery. These observations suggest that Tyr1 phosphorylation contributes to the timing of termination-factor recruitment rather than acting as a simple positive regulator of termination.

While pTyr1 participates in this conserved aspect of transcription, additional stimulus-dependent Tyr1 phosphorylation may have specialized regulatory functions. The conserved tyrosine residue provides a potential regulatory node to relay extracellular signaling events and could therefore transmit environmental cues directly to RNA polymerase II. Recent studies have identified signal-responsive kinases capable of modifying the Pol II CTD under specific biological contexts, including immediate-early gene activation and DNA-damage responses [56]. Biochemical studies demonstrated that Tyr1 phosphorylation can alter P-TEFb substrate specificity and promote Ser2 phosphorylation on CTD substrates *in vitro* [20] (Figure 1B). However, the extent to which this mechanism contributes to promoter-proximal pause release *in vivo* remains incompletely resolved. Together, these findings raise the possibility that Tyr1 phosphorylation can serve both general and context-dependent regulatory functions within the CTD code.

### pThr4 and the flanking phosphorylation paradox

Thr4 phosphorylation (pThr4) remains one of the most enigmatic modifications of the Pol II and has been termed a ‘phantom mark’ [57,58]. While the development of highly specific antibodies confirmed its existence, functional studies have painted a complex picture and have implicated Thr4 phosphorylation in multiple stages of transcription, including histone mRNA 3′-end processing, elongation, and transcription termination [59–61]. The phenotypic consequences of Thr4 perturbation vary substantially across organisms and experimental systems. For example, vertebrate CTD replacement studies revealed severe defects associated with T4A variants, whereas yeast systems generally exhibit greater tolerance to Thr4 substitution [59,60,62]. Despite the strong genetic phenotypes in vertebrates, pThr4 has historically exhibited surprisingly weak and inconsistent ChIP signals [60]. This discrepancy, together with the tolerance of Thr4 mutation in yeast, has long obscured its functional significance [63]. While mass spectrometry confirms the presence of pThr4, absolute site-specific quantification remains technically constrained by ionization suppression of multi-phosphorylated species and sequence redundancy across the CTD repeats, leaving local stoichiometry at active transcriptional sites unresolved by standard proteomics.

**Figure 2:**
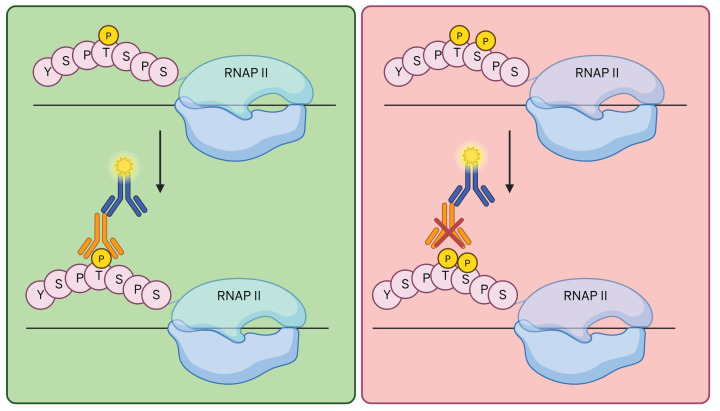
Epitope masking can obscure detection of pThr4 in the CTD Certain anti-pThr4 antibodies readily recognize phosphorylated Thr4 in the absence of neighboring modifications but exhibit reduced recognition when adjacent residues, particularly Ser5, are phosphorylated. This epitope masking can lead to under-detection of pThr4-containing CTD species despite its presence.

One potential explanation for this paradox lies in the biochemical behavior of anti-pThr4 antibodies. Although specific to pThr4, many antibodies fail to recognize pThr4 when adjacent residues within the same heptad, particularly Ser5, are phosphorylated [60] (Figure 2). This ‘masking effect,’ a phenomenon well documented in histone PTM studies [64], may also lead to systematic under-detection of combinatorial CTD marks [60,65]. Strategies designed to remove this masking (such as selective enzymatic dephosphorylation of pSer5) reveal a markedly different pThr4 landscape [59]. When the masking effect of pSer5 is removed experimentally by selective dephosphorylation, an elevated signal of pThr4 becomes detectable near transcription start sites for some of the genes, along with a secondary enrichment downstream of transcription end sites. These observations reconcile the apparent discrepancy between strong genetic phenotypes and weak antibody-based detection, suggesting that promoter-associated pThr4 has been substantially underestimated because of epitope masking.

Recognition of previously masked pThr4 near promoters also raises the possibility that pThr4 participates in combinatorial CTD signaling rather than functioning as an isolated modification. Emerging evidence further suggests that combinatorial pThr4-containing CTD states, including pThr4/pSer5 and pThr4/pSer2 species, may contribute to selective recruitment of CTD-binding factors involved in RNA processing [49,59]. For example, pThr4 can cooperate with pSer2 to enhance recognition by RPRD-family proteins, which function as scaffolds for 3′-end processing machinery [49]. Correlatively, mNET-seq and related nascent-transcription methods have also identified pThr4 enrichment near transcription termination regions, further supporting context-dependent functions for this modification [61]. Collectively, these findings suggest that the apparent ‘phantom’ nature of pThr4 reflects limitations in its detection rather than an absence of biological function and support a model in which Thr4 participates in combinatorial CTD signaling.

### Evolutionary diversification at position 7 expands CTD signaling

Among the seven residues of the consensus CTD heptad, Ser7 is unusual because it is both the least evolutionarily conserved and the most tolerant of natural variation. This apparent flexibility raises an important question: how can one of the most variable positions within the CTD contribute to conserved transcriptional regulation [52]? Various natural substitutions at this position are well-tolerated across species, a plasticity that researchers have exploited to facilitate the biochemical study of Rpb1 [49]. Although the seventh position of the CTD heptad exhibits remarkable evolutionary plasticity, constitutive phosphomimetic substitutions such as S7E are poorly tolerated, highlighting the importance of dynamic Ser7 phosphorylation rather than simply the identity of the residue [13,66,67].

Despite this tolerance for variation, Ser7 plays a sophisticated role in modulating the CTD grammar through both its side-chain identity and its phosphorylation state. *In vitro* studies indicate that pSer7 can act as a biochemical scaffold, stimulating the activity of CDK9 and CDK12 to enhance Ser2 phosphorylation [68]. This suggests a hierarchical relationship where Ser7 priming prepares the CTD for the transition into productive elongation. Furthermore, variations in this position dictate the recruitment of specific transcription regulators. Substitutions at position 7 significantly alter the binding affinities of RPRD1A and RPRD1B, potentially fine-tuning the recruitment of the dephosphorylation machinery [69]. Additionally, in yeast, pSer7 has been linked to the recruitment of the E3 ubiquitin ligase Asr1 for subtelomeric gene silencing [70]. Crucially, this recruitment depends on a ‘spatial code’ where pSer7 in one heptad must exist in tandem with pSer5 in another [70].

In addition to phosphorylation, non-consensus CTD repeats introduce lysine and arginine residues that can expand the regulatory potential of the CTD. For example, lysine acetylation at position 7 of non-consensus repeats has been linked to cross-talk with CTD phosphorylation and recognition by RPRD-family proteins (Table 1) [69]. Arginine-containing non-consensus repeats may provide additional regulatory nodes; notably, R1810 has been reported as a site of PADI2-dependent citrullination and arginine methylation [71]. This modification alters the local charge density and further expands the CTD's interactome [72]. By integrating subtle changes in residue identity with combinatorial phosphorylation patterns, the CTD significantly expands its functional repertoire, allowing for the nuanced recruitment of factors across different species and gene contexts.

## Coordination between CTD phosphorylation and histone modifications

During transcription, the CTD does more than coordinate RNA synthesis. Distinct CTD phosphorylation states also direct recruitment of chromatin-modifying complexes, establishing communication between the CTD code and the histone code [73–75]. Rather than functioning as independent regulatory systems, these two codes cooperate to coordinate transcriptional progression with restoration of chromatin structure, mediated by protein complexes that couple phosphorylated CTD states with the histone modification system.

**Figure 3:**
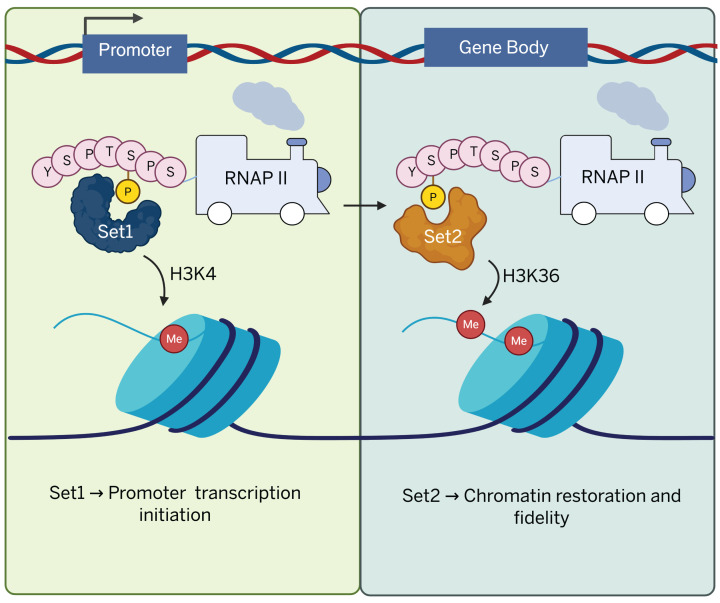
Communication between the CTD code and the histone code during transcription Distinct CTD phosphorylation states coordinate recruitment of histone methyltransferases during transcription. Promoter-associated pSer5 is associated with the recruitment of Set1 to establish H3K4 methylation, whereas pSer2-enriched elongation complexes recruit Set2 to deposit H3K36 methylation across gene bodies, helping restore chromatin organization and suppress cryptic transcription.

The best-studied example is the coordinated recruitment of Set1 and Set2 (Figure 3). In yeast, Set1/COMPASS recruitment involves interactions among phosphorylated CTD, PAF1C, and Swd2 [74,76]. Notably, the N-terminus of Set1 interacts with pSer5 of the CTD; however, Swd2 is required for optimal Set1-CTD interactions. Subsequent deletion of this region results in a global reduction of H3K4 methylation. Related but not identical mechanisms have been described for mammalian SETD1 complexes through WDR82 [25]. In addition, mammalian SETD1 complexes are targeted to CpG-rich promoters through associated CXXC domain-containing proteins that recognize unmethylated CpG DNA, providing an additional layer of recruitment beyond CTD-dependent interactions [77–79]. Conversely, yeast Set2 and mammalian SETD2 both contain SRI domains that couple H3K36 methylation to elongating Pol II, although important mechanistic differences exist between the systems [28,80]. Together, these recruitment mechanisms establish characteristic chromatin landscapes across actively transcribed genes. Promoter-proximal regions enriched in pSer5 are associated with H3K4 methylation, whereas pSer2-enriched gene bodies are accompanied by H3K36 methylation.

These histone modifications subsequently recruit downstream chromatin regulators that help restore chromatin organization following Pol II passage. In budding yeast, Set2-dependent H3K36 methylation is recognized by the Rpd3S histone deacetylase complex, which removes histone acetylation within transcribed regions and suppresses cryptic transcription initiation from internal promoters [81]. Similarly, promoter-proximal H3K4 methylation contributes to the recruitment of the Set3C histone deacetylase complex during early elongation [82]. These pathways illustrate how CTD phosphorylation patterns coordinate not only transcriptional progression but also re-establishment of chromatin integrity across active genes.

Taken together, available evidence suggests that the CTD and histone codes are increasingly recognized as highly integrated regulatory systems. As RNA polymerase II traverses a gene, changing CTD phosphorylation states recruit distinct chromatin modifiers that leave an ‘epigenetic trail’ behind the polymerase. Rather than simply marking where transcription has occurred, this trail restores chromatin organization, suppresses cryptic initiation, and preserves the chromatin environment necessary for transcriptional fidelity.

## Emerging roles of the CTD in stimulus-responsive transcription

Recent studies increasingly suggest that CTD phosphorylation is dynamically modified in response to diverse cellular stimuli [83]. Although the underlying mechanisms remain incompletely understood, accumulating evidence indicates that CTD remodeling accompanies transcriptional responses to oxidative stress, heat shock, and DNA damage. Together, these observations raise the possibility that dynamic remodeling of combinatorial CTD phosphorylation states contributes to selective transcription under changing cellular conditions.

### CTD and stress response

Acute oxidative stress induces rapid remodeling of the Pol II CTD phosphorylation landscape. Acute oxidative stress induces rapid reduction in Ser2 phosphorylation, particularly toward gene 3′ ends, disrupting productive elongation and promoting premature transcription termination [84]. Acute stress also establishes multiple RNA polymerase II stalling checkpoints, including promoter-proximal and early elongation barriers, enabling rapid global transcriptional repression before stress-responsive genes are activated [85]. At the same time, oxidative DNA lesions can directly impede RNA polymerase II translocation through a torsion-latch mechanism, further reinforcing elongation arrest [86]. These findings indicate that oxidative stress suppresses transcription through complementary mechanisms, combining physical blockage of elongating polymerase with dynamic remodeling of CTD phosphorylation to coordinate the cellular stress response [85,86].

### CTD and heat shock response

Heat shock provides another example of stimulus-dependent CTD remodeling [87,88]. Recent PRO-IP-seq analyses revealed dynamic changes in both polymerase pausing and CTD phosphorylation status during heat stress, supporting the view that stress-responsive transcription is coordinated through regulated remodeling of CTD phosphorylation states [87]. In mammalian cells, heat shock induces HSF1-dependent recruitment of P-TEFb and other elongation factors to activate stress-responsive genes [89,90]. In yeast, recent genome-wide studies have revealed extensive reprogramming of RNA polymerase II occupancy and associated transcription complexes, with coordinated depletion from housekeeping genes and enrichment at stress-responsive loci [91,92].

### CTD and DNA repair

CTD phosphorylation also contributes to genome maintenance through CDK12/13-dependent regulation of DNA damage response genes [93,94]. CDK12 promotes productive elongation across many transcription units and influences global CTD phosphorylation patterns [95,96]. Notably, long DNA damage response genes, including BRCA1 and BRCA2, are particularly sensitive to CDK12 perturbation because the maintenance of full-length transcription is required across these unusually long units [93,94]. Current models suggest that this sensitivity arises not only from effects on elongation processivity but also from increased utilization of premature cleavage and polyadenylation sites within long transcription units [97]. Consequently, the activities of CDK12 and, in some contexts, CDK13, help maintain full-length transcription of a subset of genes that are critical for genome maintenance and homologous recombination, bridging the gap between the act of transcription and the maintenance of genomic integrity [95].

Although these biological contexts differ substantially, they share a common theme: CTD phosphorylation is dynamically remodeled in response to changing cellular conditions, suggesting that combinatorial CTD states contribute not only to the transcription cycle itself but also to adaptive gene regulation.

## Concluding remarks

While many individual CTD phosphorylation events and binding interactions have been experimentally characterized, how these modifications function collectively at the systems level remains less well understood.

Here, we outline an emerging conceptual framework that integrates recent biochemical, structural, and genomic observations into a broader model for CTD-mediated transcriptional regulation. Rather than serving solely as a platform for factor recruitment, the CTD may encode a combinatorial regulatory grammar through which RNA polymerase II integrates transcriptional, signaling, and chromatin-derived inputs. A major challenge for the field will be determining how combinations of CTD modifications are generated, interpreted, remodeled, and removed during distinct transcription programs. Future studies will need to move beyond individual phosphorylation sites to understand how combinatorial CTD states contribute to the selective regulation of housekeeping genes, developmental programs, stress-response, and DNA repair.

Beyond their mechanistic significance, CTD regulatory pathways may also provide new opportunities for therapeutic intervention. Because many diseases are associated with altered transcriptional programs, a deeper understanding of CTD-dependent signaling networks may reveal strategies for selectively modulating transcriptional outputs. More broadly, emerging evidence suggests that CTD phosphorylation can influence not only factor recruitment but also the physical organization of transcriptional machinery through condensate formation and remodeling. Together, these observations support a model in which the CTD serves as an important regulatory interface linking transcription, cellular signaling, and chromatin state.

## Perspectives

- The RNA polymerase II CTD is fundamental to eukaryotic life, acting as the primary coordinator that integrates transcription progression, RNA processing, and genome stability.
- Recent structural and mass spectrometry advances have continued shifting the paradigm, supporting the idea that the CTD is a dynamic, combinatorial signaling processor that communicates with the histone code and interprets environmental stress.
- The next research frontier lies in decoding the complex, multivalent ‘CTD grammar’ at a systems level and exploiting phase separation properties or elongation kinetics as novel therapeutic vulnerabilities in oncology.

## Open Access

Open access for this article is provided via our Subscribe to Open programme, made possible through the support of our subscribing institutions. Learn more here: https://portlandpress.com/pages/open_access_options_and_prices#conditional.

## Abbreviations

CTD: C-terminal domain
PIC: pre-initiation complex
Pol II: RNA polymerase II
pThr4: Thr4 phosphorylation
